# Progesterone receptor membrane component 2 regulates the neuronal activity and participates in epileptic seizures in experimental mice

**DOI:** 10.1002/ibra.12088

**Published:** 2023-01-11

**Authors:** Xiaoyan Yang, Wenbo Lv, Yong Yang, Juan Yang, Haiqing Zhang, Zucai Xu

**Affiliations:** ^1^ Department of Neurology Affiliated Hospital of Zunyi Medical University Zunyi China; ^2^ Division of Clinical Neuroscience Chiba University Center for Forensic Mental Health Chiba Japan; ^3^ Collaborative Innovation Center of Tissue Damage Repair and Regeneration Medicine of Zunyi Medical University Zunyi Guizhou China

**Keywords:** astrocytes, epilepsy, hippocampus, neurons, progesterone receptor membrane component 2

## Abstract

It was found the expression of progesterone receptor membrane component 2 (PGRMC2) in the histone of epileptic mice was lower than that of normal mice. In this study, we found by the immunofluorescence technique, PGRMC2 was expressed in both astrocytes and neurons of the mouse hippocampus. In addition, the seizure latency and seizure grade of mice in each group were observed after stereotactic injection of the PGRMC2 knockdown virus, PGRMC2 overexpression lentivirus, and related null virus into the hippocampus of mice. It was found that the seizure latency of mice in the PTZ + siPGRMC2 group was prolonged compared with the null virus group. The seizure latency was shortened in the PTZ + PGRMC2 group. The number of grade IV and above seizures in the PTZ + siPGRMC2 group was significantly reduced, while the number of grade IV and above seizures in the PTZ + PGRMC2 group was significantly increased. It was found that the nerve cells in the PTZ + siPGRMC2 group were still intact. In the PTZ + PGRMC2 group, the neural cells were damaged, the intercellular space was widened, and the number of cells was reduced. These findings support that PGRMC2 may be involved in epileptic seizures.

## Graphical abstract

To explore the possible mechanism of the role of PGRMC2 in epileptic seizures. In conclusion, combined with the results of this study, PGRMC2 can colocalize with astrocytes and neurons, and intervention of PGRMC2 expression can have a regulatory effect on neuronal function and seizure.

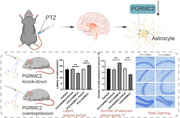

## 1 INTRODUCTION

Epilepsy is a clinical syndrome caused by abnormal excessive and synchronous discharge activity of brain neurons. It is a chronic and persistent encephalopathy caused by multiple causes, characterized by recurrent, transient, repetitive, and stereotypic clinical seizures.^1^, ^2^ It is the second‐largest disease in neurology after stroke.^3^ According to the World Health Organization (WHO), there are about 70 million epilepsy patients worldwide^4^, ^5^ and more than 9 million in China, with a prevalence rate of 4‰–7‰, which is increasing by 400,000 people annually. The prevalence of active epilepsy is 4.6%. About 30% of patients have intractable epilepsy, and the mortality rate is 1.3–3.6 per 100,000, which is 2–3 times the general population.^6^, ^7^, ^8^, ^9^ Due to its high incidence, recurrent, refractory, and social discrimination, it has burdened the patient's body, mind, family, and society^10^, ^11^, ^12^ and has been listed as one of the key neuropsychiatric diseases for prevention and treatment by WHO.

With the advancement of science, technology, and medical technology, there has been significant progress in the in‐depth study of epilepsy pathogenesis, the clinical application of new drugs and technology, and the clinical treatment of epilepsy. However, because of the diversity of its clinical etiology and the complexity of its pathogenesis, we continue to face enormous challenges, and it is unclear what is the primary cause of its clinical effect. At present, research on its pathogenesis include neurotransmitters,^13^ ion channels,^14^ neuroglial cells, neuronal damage, energy metabolism, synaptic connection, genetic and immune abnormalities, and neural network dysfunction,^15^, ^16^, ^17^, ^18^ which are involved in the initiation, propagation, and termination of epileptic discharge. Studies related to the occurrence and seizure mechanism of epilepsy are particularly important for developing epilepsy prevention and treatment strategies, among which the mechanism of neuronal injury has been one of the focuses of epilepsy‐related research.^19^ Frequent seizures may cause severe and irreversible pathological changes in the brain.^20^ Although active control of epileptic seizures is very important in clinical treatment, the consequences of recurrent seizures, such as neuronal damage, should not be ignored. Neuronal injury results from recurrent seizures and may also be the potential cause of recurrent seizures.^21^, ^22^ Therefore, reducing neuronal injury after epileptic seizures and actively preventing brain injury are of great clinical value.

The membrane‐associated progesterone receptor family proteins (MAPRs) family includes four members: progesterone receptor membrane component protein 1 (PGRMC1), PGRMC2, neurotrophic factor protein (Neustein), and cytochrome B5 domain protein (CYB5D). It is an evolutionarily conservative membrane receptor protein and plays an important role in cytoskeleton rearrangement, cell proliferation, cell survival, apoptosis, and chemosensitivity. PGRMC1 and PGRMC2 have been found in the uterus, fat, cerebral cortex, hippocampus, and other parts.^23^ PGRMC1 and PGRMC2, as nonclassical signaling molecules, mediate various processes such as progesterone neuroprotection and neuroplasticity.^24^ PGRMC2 is a newly discovered small‐molecule protein. There is very little research on the function of PGRMC2. Three hundred and one articles on the “progesterone receiver membrane component” were retrieved from Pubmed, and the related research mainly involves “tumor, metabolism, and progesterone.” However, whether PGRMC2 participates in epileptic seizures and whether it has neuroprotective effects have not been reported.

In this study, PGRMC2 overexpression lentivirus and PGRMC2 knockdown lentivirus were injected into mice by stereotactic injection, and then, a mouse model of chronic epilepsy was constructed using pentotetrazolium (PTZ) to explore its possible role in epileptic seizures and neuronal damage, providing a new research direction for exploring the target of new antiepileptic drugs.

## 2 MATERIALS AND METHODS

### 2.1 Animals

Healthy adult male C57BL/6 J mice (weight 22–25 g, specific pathogen free) were selected. The experimental animals were fed in a 12 h light/dark environment, with an ambient temperature (22–26°C), autonomous eating and drinking, and separate cage feeding (5 mice/cage). The experimental animals were provided by Hunan Slake Jingda Laboratory Animal Co., Ltd. (License number: SCXK (Xiang) 2019‐0004). Ninety mice were randomly divided into six groups (*n* = 15): normal mice group (CON), epilepsy group, PGRMC2 overexpression null virus group (PTZ + Vector group), PGRMC2 knockdown null virus group (PTZ + siCtrl group), PGRMC2 overexpression group (PTZ + PGRMC2 group), and PGRMC2 knockdown group (PTZ + siPGRMC2 group). All animal operations followed the ethics code and were approved by the Ethics Committee (Ethical review number: KLLY (A)–2020‐037).

### 2.2 Construction of the PTZ chronic epilepsy model

The susceptibility to seizures was gradually reduced by intraperitoneal subthreshold injection of PTZ on alternate days, and eventually, generalized tonic‐clonic seizures (GTCS) were developed as the number of injections increased. The mice were intraperitoneally injected with a subthreshold dose of PTZ (35 mg/kg) every 48 h. After each injection of PTZ, the seizures of mice were observed for 30 min. Chronic ignition was considered successful in mice with three or more consecutive seizures of grade 4 or 5. If the seizure did not end within 5 min, the seizure was terminated by intraperitoneal injection of diazepam (5 mg/kg).

### 2.3 Construction, amplification, and lentivirus packaging of plasmids

We used a modified second‐generation lentivirus packing system from GeneChem. The system is composed of a packaging plasmid (pHelper 1.0) encoding gag, pol, and rev; an envelope plasmid expressing VSV‐G (pHelper 2.0); and a transfer plasmid encoding the transgene (GV series). The GV492 plasmid carrying PGRMC2 cDNA under the CBh promoter was used for ectopic expression, with an empty GV492 plasmid (vector) as the control. For gene silencing, the GV493 plasmid bearing the shRNA targeting PGRMC2 mRNA (NM_027558.1, 621‐639) under the human U6 promoter (SiPgrmc2) was used, with GV492 containing a scramble shRNA (SiNC) as the control.

Twenty‐four hours before transfection, 5 × 10^6^ 293 T cells were passaged into a 10 cm Petri dish containing 10 ml of Dulbecco's Modified Eagle Medium (DMEM) plus 10% fetal bovine serum and cultured at 37°C, with 5% CO_2_. Two hours before transfection, cells were adapted to a serum‐free medium.

For a single lentivirus packing, plasmid solutions (GV series vector plasmid 20 μg, pHelper1.0 vector plasmid 15 μg, pHelper2.0 vector plasmid 10 μg) were prepared in a sterilized centrifuge tube, mixed well with the corresponding volume of GK transfection reagent, adjusted the total volume to 1 ml, and incubated at room temperature for 15 min. The mixture solution was added dropwise to the 293 T cell plate, finely mixed, and cultured in a 37°C, 5% CO_2_ cell incubator.

Six hours after transfection, the medium containing the transfection mixture was discarded. The cells were gently washed with PBS, refed with 10 ml of DMEM plus 10% fetal bovine serum, and continued to be cultured at 37°C and 5% CO_2_ for 48–72 h until harvesting lentiviruses.

### 2.4 Intervention in animal experiments

The PTZ + PGRMC2 group, PTZ + Vector group, PTZ + siPGRMC2 group, and PTZ + siCtrl group were constructed. The above four lentiviruses were injected by hippocampal stereoscopic positioning. The anterior tantanel was the base point, 2.0 mm backward, ±1.5 mm from the left and right side of the median line, the skull was drilled with a miniature electric drill according to the corresponding coordinates, the skull was held and fixed on the locator with 2 μl microinjection cannula, and the lentivirus was sucked into the hole vertically and slowly to the depth of 1.5 mm. After 2 weeks of routine feeding, Western blot analysis (WB) was used to measure the transfection efficiency of the lentiviral vector. The remaining mice were treated as an epilepsy model group.

### 2.5 Preparation of brain tissues

#### 2.5.1 Immunofluorescence and Nissl staining

All the mice were anesthetized with 1% pentobarbital sodium and then killed. After taking out the brain tissue, all the brain tissues were labeled and put into 4% paraformaldehyde for overnight fixation in a 4°C refrigerator. These brain tissues were sent to the pathology department for paraffin embedding. Following these steps, immunofluorescence staining and Nissl staining were performed.

#### 2.5.2 WB analysis

The mouse brain tissue was taken out according to the above method, placed on ice to quickly and accurately separate the hippocampus tissue, and stored in the −80°C refrigerator for WB detection.

### 2.6 WB

Proteins from the hippocampus were collected in Radio‐Immunoprecipitation Assay buffer. 12.5% SDS polyacrylamide gel electrophoresis was configured according to the molecular weight of the protein. After completing electrophoretic separation and transferring the gel onto PVDF membranes, the polyvinylidene fluoride (PVDF) membranes were blocked with treated 5% skimmed milk powder for 1 h and incubated overnight with primary antibodies. PGRMC2 polyclonal rabbit Antibody (1:500, 1 mg/ml, Signalway Antibody) was used as the primary antibody. After washing the PVDF membrane treated above, specific binding was performed with a secondary antibody (1:5000, Sanying) conjugated with horseradish peroxidase. They were then stained with an ECL chemiluminescence kit and imaged with an electrophoretic gel imaging analysis system.

### 2.7 Immunofluorescence

First, the slides of each group were dewaxed, and then, the slices were placed in preheated Ethylene Diamine Tetraacetic Acid (EDTA) antigen repair buffer in a microwave oven on low fire for antigen repair. After that, the sections were placed in 3% hydrogen peroxide solution to block endogenous peroxidase, and then, the biotin‐avidin system was added to drop for 30 min. The first primary antibody (NEUN rabbit polyclonal antibody 1:300 Servicebio) was added by drop, and the slices were placed flat in a wet box and incubated overnight at 4°C in a refrigerator. The corresponding HRP‐labeled secondary antibody (1:500, Sanying) was added and incubated for 50 min at room temperature. Then, FITC‐TSA (1:300 Servicebio, Wuhan, China) was dropped onto the sections. The sections were placed in a microwave oven on low heat for antigen repair using a prepared EDTA antigen repair buffer. Another primary antibody (GFAP chicken polyclonal antibody 1:500 m, Servicebio, Wuhan, China) was added, and the sections were placed flat in a wet box and incubated overnight at 4°C in a refrigerator. The corresponding HRP‐labeled secondary antibody (1:500, Sanying) was dropped and incubated for 50 min at room temperature in the dark. The sections were placed in a microwave oven on low heat for antigen repair after adding 647‐TSA. The third primary antibody (PGRMC2 Polyclonal mouse Antibody 1:50, Sanying) was added, and the slices were placed flat in a wet box and incubated overnight at 4°C in a refrigerator. The Cy3‐labeled fluorescent secondary antibody (1:300, Servicebio) was added and incubated for 50 min at room temperature in the dark. An autofluorescence quencher was added for 5 min, and the slices were gently dried. Then, the slices were sealed with an anti‐fluorescence quencher and covered with a glass of the corresponding size. The sections were placed under a scanner for image acquisition (Olympus positive fluorescence microscope).

### 2.8 Nissl's staining

First, the slides of each group were dewaxed. CresyI Violet Stain (Beijing Leagene Biotechnology Co,. Ltd) was added to the slides to cover the tissues fully, and the slides were placed in a wet box and stained in a constant temperature oven at 56°C for 1 h. Nissl differentiation was added for 1 min, and the slides were immersed in absolute ethanol for rapid dehydration for 5 s. The slides were immersed in xylene I and II for 1 min each. After the tissue was fully covered with neutral resin, the tissue was placed under the Orthostatic optical microscope (NIKON ECLIPSE C1) to observe the tissue morphology. (Reagents used for Nissl staining were purchased from Beijing Regen Biotechnology Co., Ltd.).

### 2.9 Statistical analysis

BM® SPSS® Statistics 18.0 software (Armonk) was used for the statistical data of this study, and GraphPad Prism (San Diego) 8.0 software was used for making icons. Normality testing was performed at first, and the experimental data conforming to normal distribution were represented by mean ± standard deviation (SD). WB results: A *T*‐test was used for comparison between two groups, one‐way analysis of variance was used for comparison between multiple groups, *p* < 0.05, and the difference was statistically significant. Behavioral results: one‐way analysis of variance was used for comparison between multiple groups, *p* < 0.05, and the difference was statistically significant.

## 3 RESULTS

### 3.1 Expression of PGRMC2 in the hippocampus of normal and chronic epilepsy mice

**Figure 1:**
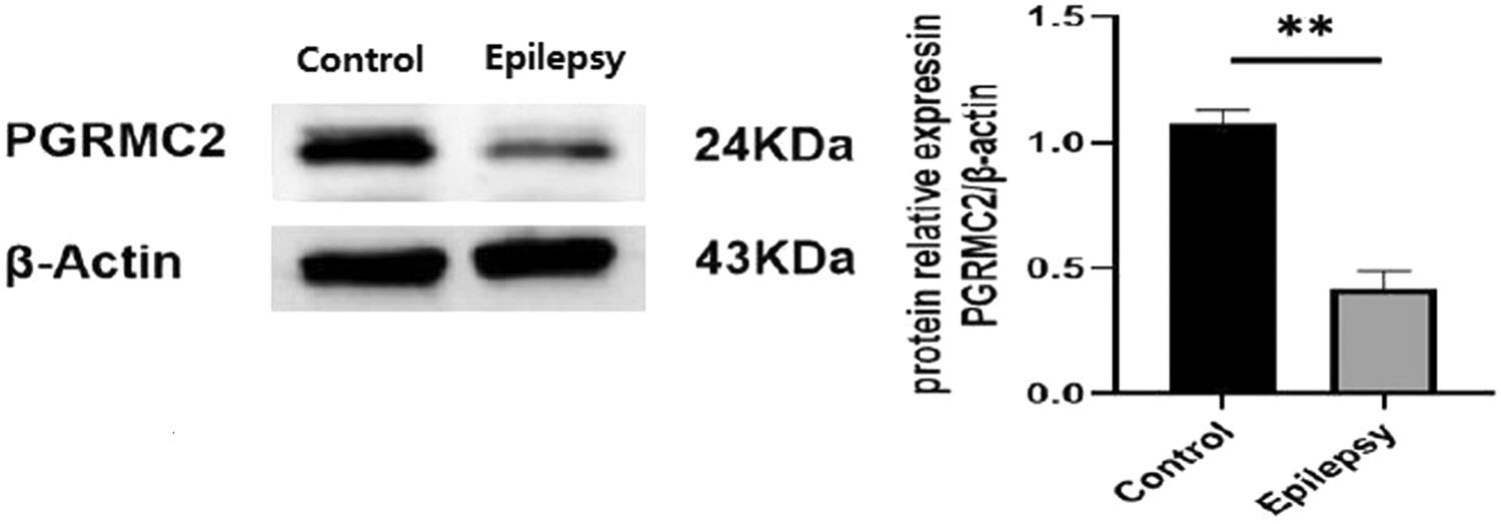
Expression of PGRMC2 in the hippocampus of normal and chronic epileptic mice. The results revealed that the expression of PGRMC2 of the hippocampus in the chronic epilepsy mouse model was significantly reduced than that in the control group (*n* = 3, **p < 0.01).

In this part of the experiment, we used PTZ to induce the chronic epilepsy model. A total of 15 mice were injected with pentylenetetrazol and more than 80% of mice in each group were successfully modeled. There was no seizure in the saline injection group. We used WB technology to detect the protein expression of PGRMC2 in the hippocampus of CON and chronic epilepsy model mice. The results showed that compared with CON, the protein expression of PGRMC2 in the chronic epilepsy mouse model was significantly reduced (*p* < 0.01) (Figure 1).

### 3.2 Distribution and localization of PGRMC2 in normal and epilepsy group mouse brain tissues

**Figure 2:**
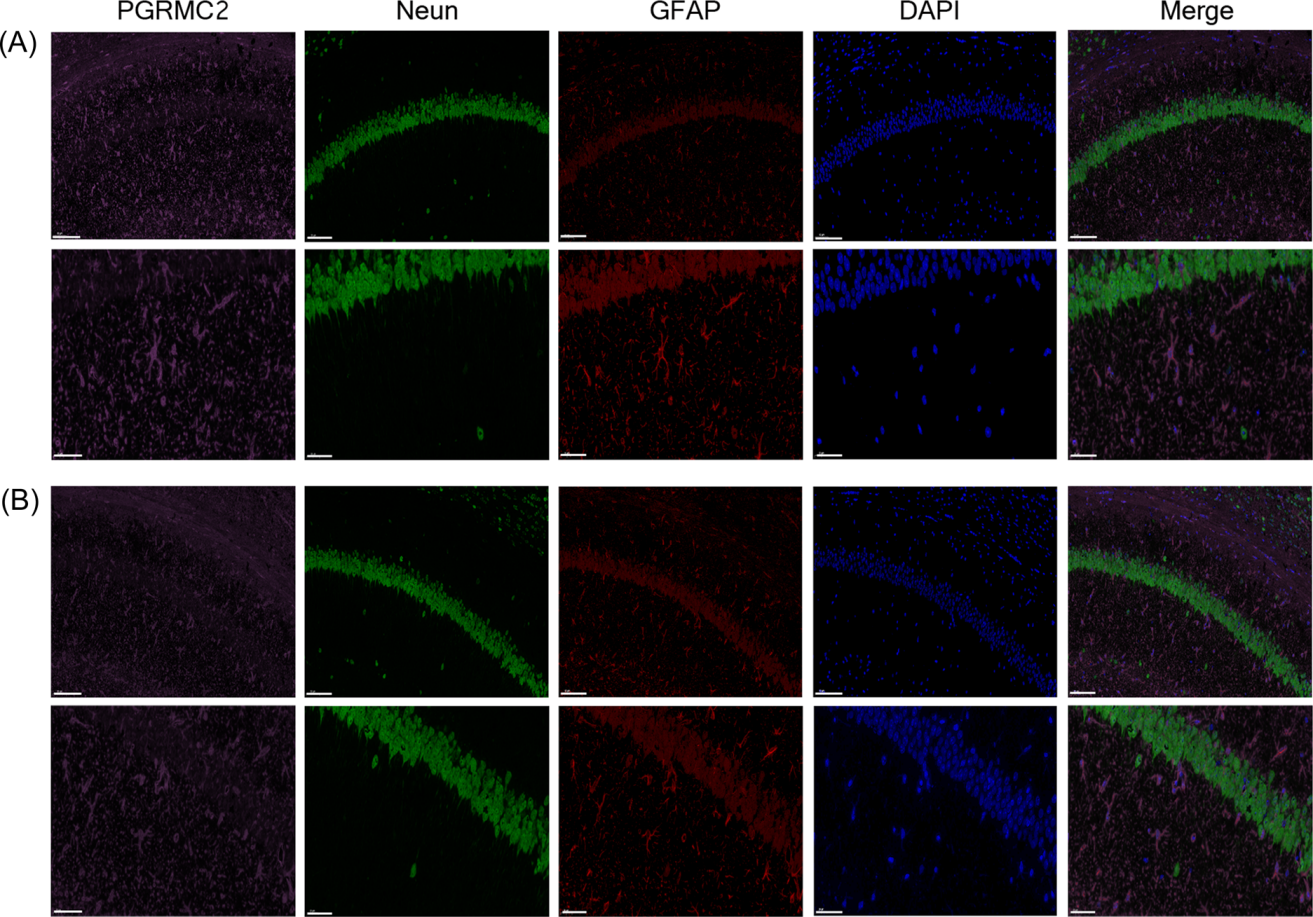
Distribution and localization of PGRMC2 in the mice brain tissue of normal and chronic epileptic mice. The following figure is a partial enlargement of AB (The scale bar = 100 μm). (A) In the mice brain tissue of norma, PGRMC2 (purple), colocalized with GFAP (red) and Neun (green) (*n* = 3). (B) In the mice brain tissue of chronic epileptic mice, PGRMC2 (purple) colocalized with GFAP (red) and Neun (green) (*n* = 3). (The scale bar = 100 μm). [Correction added on 17 March 2023, after first online publication: The figure 2 was revised in this version for better clarity.] [Color figure can be viewed at wileyonlinelibrary.com]

We detected the distribution and localization of PGRMC2 in CON and epilepsy group brain tissues by the immunofluorescence technique. The results showed that PGRMC2 was expressed in both astrocytes and neurons of the mouse hippocampus (Figure 2).

### 3.3 Evaluation of PGRMC2 lentiviral vector effectiveness

**Figure 3:**
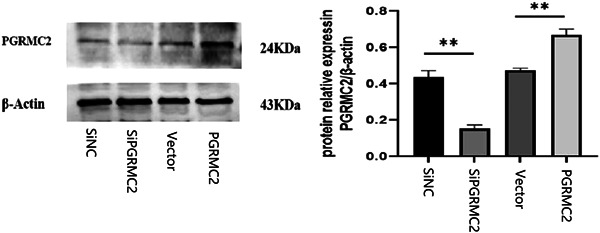
Evaluation of PGRMC2 lentiviral vector effectiveness. The protein expression of the PTZ + siPGRMC2 group was significantly decreased after lentivirus intervention, while the histone expression of the PTZ + PGRMC2 group was significantly increased. (*n* = 3, **p < 0.01). PTZ + PGRMC2 + Vehicle = PTZ + Vector = Vector. PTZ + PGRMC2 − Vehicle = PTZ + siCtrl = siCtrl. PTZ + PGRMC2 + = PTZ + PGRMC2 = PGRMC2. PTZ + PGRMC2− = PTZ + siPGRMC2 = siPGRMC2.

We injected the PGRMC2 knockdown lentivirus, PGRMC2 knockdown null lentivirus, PGRMC2 overexpression lentivirus, and PGRMC2 overexpression null lentivirus by stereo localization. After 2 weeks, the protein expression of PGRMC2 in the hippocampus of each group of mice was detected by the WB technology. The results showed that the protein expression of the PTZ + siPGRMC2 group was significantly decreased after lentivirus intervention, while the protein expression of the PTZ + PGRMC2 group was significantly increased, which confirmed the effectiveness of lentiviral transfection (*p* < 0.01) (Figure 3).

### 3.4 Effect of PGRMC2 on seizure latency and grand mal seizure times in mice

**Figure 4:**
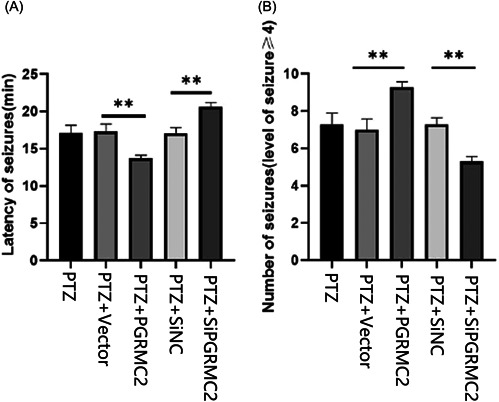
Effects of PGRMC2 on the latency of epileptic seizures and the number of grand seizures in mice. (A) Effect of intervening PTZ + PGRMC2 + on the latency of epileptic seizures in mice (*n* = 7; **p < 0.01). (B) Effect of intervening PTZ + PGRMC2 + on the number of grand seizures in mice (*n* = 7, **p < 0.01). Epilepsy group = PTZ. PTZ + PGRMC2 + Vehicle = PTZ + Vector = Vector. PTZ + PGRMC2 − Vehicle = PTZ + siCtrl = siCtrl. PTZ + PGRMC2 + = PTZ + PGRMC2 = PGRMC2. PTZ + PGRMC2− = PTZ + siPGRMC2 = siPGRMC2.

Epilepsy group, PTZ + Vector group, PTZ + siCtrl group, PTZ + PGRMC2 group, and PTZ + siPGRMC2 group seizure latency time was 17.10 ± 0.98, 17.32 ± 0.90, 17.05 ± 0.69, 13.72 ± 0.36n, and 20.63 ± 0.51 min, respectively (Table 1). The results showed that the seizure latency of mice in the PTZ + siPGRMC2 group was significantly longer than that in the PTZ + siCtrl group (*p* < 0.01). However, the PTZ + PGRMC2 group was significantly shorter than the PTZ + Vector group (*p* < 0.01). There was no significant difference among the epilepsy group, PTZ + Vector group, and PTZ + siCtrl group (*p* > 0.05). The number of grand seizures greater than or equal to grade 4 of the epilepsy group, PTZ + Vector group, PTZ + siCtrl group, PTZ + PGRMC2 group, and PTZ + siPGRMC2 group during the construction of the pentatetrazolium chronic epilepsy model was 7.29 ± 0.56, 7.00 ± 0.54, 7.29 ± 0.33, 9.29 ± 0.26, and 5.29 ± 0.26, respectively (Table 1). The results show that, compared to the PTZ + siPGRMC2 group, the number of grand seizures of mice in the PTZ + PGRMC2 group was significantly  (*p* < 0.01). Compared to the PTZ + Vector group, the number of grand seizures of mice in the PTZ + PGRMC2 group was significantly increased (*p* < 0.01). There was no significant difference among the epilepsy group, PTZ + Vector group, and PTZ + siCtrl group (*p* > 0.05) (Figure 4).

**Table 1:** Effects of PGRMC2 on the latency of epileptic seizures and the number of grand seizures in mice

Groups	Latency of seizures (min)	Number of seizures (level of seizure ≥ 4)
Epilepsy	17.10 ± 0.98	7.29 ± 0.56
PTZ + PGRMC2 + Vehicle	17.32 ± 0.90	7.00 ± 0.54
PTZ + PGRMC2 − Vehicle	17.05 ± 0.69	7.29 ± 0.33
PTZ + PGRMC2+	13.72 ± 0.36	9.29 ± 0.26
PTZ + PGRMC2−	20.63 ± 0.51	5.29 ± 0.26

### 3.5 Nissl staining results

**Figure 5:**
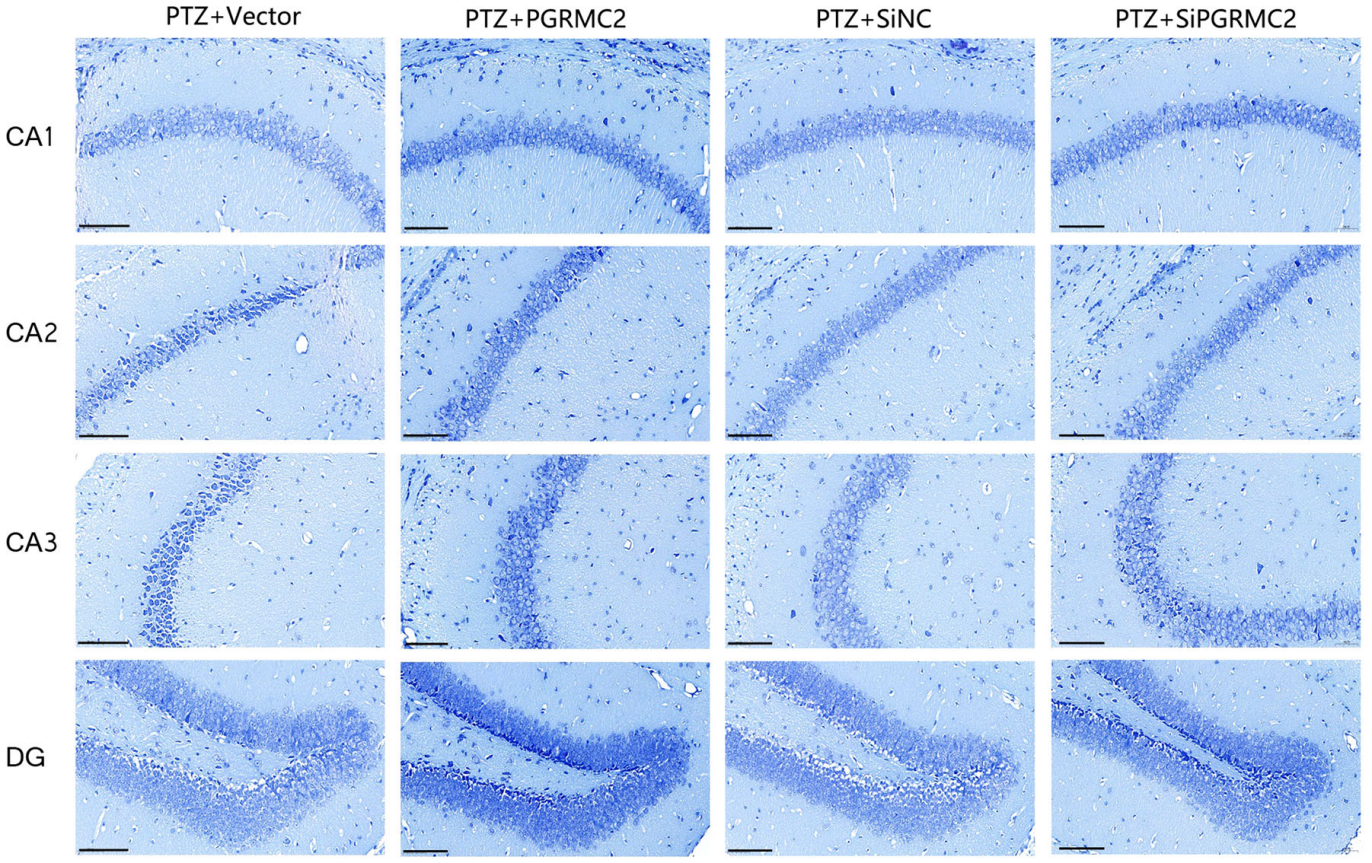
Effects of PGRMC2 with different expressions on neurons. Neuronal damage in the PTZ + siPGRMC2 group was not obvious in the process of long‐term epileptic seizures. In the PTZ + PGRMC2 group, neurons were seriously damaged in the process of long‐term epileptic seizures in mice. The damage degree of nerve cells in the PTZ + Vector group and siCtrl group was slightly better than in the PTZ + PGRMC2 group. (The scale bar = 100 μm). PTZ + PGRMC2 + Vehicle = PTZ + Vector = Vector. PTZ + PGRMC2 − Vehicle = PTZ + siCtrl = siCtrl. PTZ + PGRMC2 + = PTZ + PGRMC2 = PGRMC2. PTZ + PGRMC2− = PTZ + siPGRMC2 = siPGRMC2. [Color figure can be viewed at wileyonlinelibrary.com]

By observing the hippocampal CA1, CA2, CA3, and DG regions of mice in each group, we found that nerve cells in the PTZ + siPGRMC2 group were still intact with relatively dense and regular cell arrangement, clear nucleolus, and neat boundary, indicating that neuronal damage in this group was not obvious in the process of long‐term epileptic seizures. These results further indicate that PGRMC2 knockdown intervention has a neuroprotective effect. However, nerve cells in the PTZ + PGRMC2 group were seriously damaged, with the disordered arrangement, widened intercellular space, reduced cell number, and relatively irregular morphology, indicating that after PGRMC2 overexpression intervention, neurons were seriously damaged in the process of long‐term epileptic seizures in mice, resulting in excessive apoptosis of nerve cells. The damage degree of nerve cells in the PTZ + Vector group and the PTZ + siCtrl group was slightly better than the PTZ + PGRMC2 group (Figure 5).

## 4 DISCUSSION

Epilepsy is a chronic disease characterized by sudden abnormal brain neuron discharge, leading to transient brain dysfunction. It has been listed as one of the key neuropsychiatric diseases for prevention and treatment by WHO. Increased excitability of neurons is the basis of epileptic seizures.^25^ Astrocytes are a group of glial cells in the central nervous system.^26^ Astrocytes and neurons play a key role in regulating normal brain function, including maintaining ion and neurotransmitter homeostasis, metabolism, the regulation of neuroinflammation, synaptic development, and signaling,^27^, ^28^, ^29^, ^30^ which are significantly disrupted in epilepsy. Studies have shown that astrocytes are associated with epileptogenesis and seizure‐mediated brain damage.^15^, ^31^ Studies have found that astrocytes can participate in and promote epileptogenesis through various mechanisms, such as increased gap junction coupling, impaired glutamate transporter function, and blood–brain barrier disruption.^32^, ^33^ Therefore, astrocyte injury and epileptogenesis may be causal to each other. This study found that PGRMC2 was colocalized with astrocytes and neurons in the normal mouse hippocampus. However, does colocalization of the two regulate seizures? By observing the behavior of experimental mice, we found that the knockdown of PGRMC2 significantly prolonged the seizure latency and reduced the number of grand seizures, while PGRMC2 overexpression shortened the seizure latency and increased the number of grand seizures in mice. Nonetheless, through what mechanism is PGRMC2 involved in the regulation of seizures?

It was found that PGRMC1 was expressed in astrocytes and neurons. Studies have found that in animal models of traumatic brain injury, the expression level of PGRMC1 is increased in brain edema tissues and expressed in astrocytes. This study speculated that PGRMC1 might play a neuroprotective role by reducing brain edema.^34^ In addition, it has also been reported that PGRMC1 may participate in menstrual seizures through neuroprotection and neural plasticity. PGRMC2 is highly homologous with PGRMC1, and highly homologous proteins have many common biological characteristics, such as PGRMC2 and PGRMC1 have similar Cyt‐b5 domains and their biological activity largely depends on the binding of heme with their Cyt‐b5‐like heme/steroid‐binding domain.^35^ In this study, PGRMC2 was co‐localized with astrocytes and neurons in the mouse hippocampus. Chan et al.^36^ conducted neuropathological studies on patients with mitochondrial epilepsy and found all the patients had a common feature that astrocytes were involved. The glutamine synthetase deficiency in astrocytes of patients can lead to mitochondrial diseases, and the decrease of GABA in brain tissues while causing energy metabolism disorder may cause seizures. During the interval between seizures, the energy metabolism may be reduced due to the loss of local neurons, cortical atrophy, and decreased synaptic activity.^37^ On the contrary, during epileptic seizures, the highly abnormal synchronized discharge of neurons requires a large amount of ATP, which hinders the glucose transport and oxidative metabolism process, leading to a reduction in ATP production, resulting in the impairment of neuronal ion transport, neurotransmitter uptake and release, and the obstruction of the signal transmission process, which ultimately leads to epilepsy.^38^ Nature^39^ reported the latest research findings that PGRMC2 is highly expressed in adipocytes. It is a receptor protein necessary for free heme and signal pathway heme to pass to the nucleus. In brown fat with high demand for heme, the loss of PGMRC2 will cause changes in gene expression, resulting in serious mitochondrial defects and eventually leading to energy metabolism disorders. When mice eat a high‐fat diet, they will have serious metabolic disorders, making mice unable to maintain their body temperature, suggesting that PGRMC2 may be a protein closely related to cell energy metabolism. The balance of energy metabolism is crucial for regulating the over‐excitation of neuronal networks and maintaining the normal function of neurons.^40^ Studies have shown that excessive abnormal neural activity occurs when neurons have sufficient energy sources; on the contrary, energy deprivation can be used as an intrinsic mechanism to terminate seizures during their occurrence.^41^ In animal models of epilepsy, astrocytes show significantly enhanced Ca2+ signaling,^42^ which further promotes the secretion of glial transmitters, such as glutamate and adenosine.^43^ However, glutamatergic signals from astrocytes can trigger hippocampal synaptic transmission, leading to long‐term enhancement or inhibition, thereby activating the postsynaptic excitability of neurons.^44^ On the other hand, astrocyte‐derived adenosine has been shown to activate presynaptic A1 or A2A receptors in neurons to regulate synaptic transmission significantly, thereby affecting neuronal function.^44^, ^45^ In addition, astrocytes can also release many neuroactive substances, such as prostaglandins and TNF‐α, which also have regulatory effects on the development and function of neurons.^46^ However, the occurrence of epilepsy may be related to a variety of factors, including genetic susceptibility, developmental dysfunction, and nerve damage,^47^, ^48^ which may lead to synaptic morphological changes and highly excitatory neuron transmission.^49^ In addition, nerve damage caused by traumatic brain injury, hypoxia, or thermal convulsions may be associated with neuronal death, dysfunctional synaptic modification, and the generation of hyper‐excitatory networks, which may also lead to spontaneous seizures.^50^ At the same time, chronic seizures can cause changes in the hippocampal microenvironment,^51^ and neurotoxicity caused by epilepsy may lead to neuronal damage in the hippocampal region.^52^

In conclusion, combined with the results of this study, PGRMC2 can colocalize with astrocytes and neurons, and intervention of PGRMC2 expression can have a regulatory effect on neuronal function and seizure. Therefore, we speculate that PGRMC2 is involved in the occurrence and development of epileptic seizures, but the mechanism through which PGRMC2 affects the occurrence and development of epileptic seizures is unknown and needs further exploration.

## AUTHOR CONTRIBUTIONS

**Xiaoyan Yang**: Conceptualization; methodology; formal analysis; visualization; writing original draft. **Wenbo Lv**: Conceptualization; methodology; formal analysis; visualization; writing original draft. **Yong Yang**: Methodology; prepared figures and revision of manuscript. **Juan Yang**: Data curation; prepared tables. **Haiqing Zhang**: Funding acquisition; prepared figures. **Zucai Xu**: Conceptualization; supervision; writing review and editing. All authors reviewed the manuscript. All authors read, revised, and approved the final manuscript.

## CONFLICT OF INTEREST

The authors declare no conflict of interest.

## ETHICS STATEMENT

All procedures were performed per the Guide for and use of Medical laboratory animals (Ministry of Health of China, 1998) and approved by the animal experiment ethics committee of Zunyi Medical College (Ethical review number: KLLY (A)–2020‐037).

## Data Availability

The authors confirm that the data of this study are available within the article.
